# Sexual health needs data – and ideas for shaping a positive sexual culture!

**DOI:** 10.25646/9954

**Published:** 2022-06-29

**Authors:** Petra Kolip

**Affiliations:** Bielefeld University, School of Public Health

Sexual health and well-being are rarely a public health issue. It is therefore all the more important to persistently draw attention to this issue. The topic is multifaceted and cannot be reduced to sexually transmitted diseases. The WHO definition of sexual health from 2015 establishes a close connection to general well-being: “Sexual health is an integral part of overall health, well-being and quality of life. It is a state of physical, emotional, mental and social well-being in relation to sexuality and not merely the absence of disease, dysfunction or infirmity.” [1]. Sexual health is closely linked to human rights and implies the possibility of having pleasurable and safe sexual experiences, free of coercion, discrimination and violence [1]. Studies on so-called integrated biological and behavioural surveillance, which are an important basis for interventions, have been conducted at the Robert Koch Institute for many years. These studies combine data on the frequency of certain infectious diseases with data on sexual behaviour. Vulnerable groups are also addressed: Men who have sex with men, drug users (intravenous), sex workers and migrants. Some of the articles in this issue of the Journal of Health Monitoring address other important topics, such as the sexual and contraceptive behaviour of adolescents, the use of different sources of information on sexuality education and abortions.

Hintzpeter’s et al. article is based on data from KiGGS Wave 2 and shows that adolescents are becoming sexually active later and later, that in young adulthood sexuality is predominantly lived in stable couple relationships and that condom and pill are still the most important contraceptives. It will be exciting to observe how sexual and contraceptive behaviour has changed here as a result of the Corona pandemic. Even if contraceptive behaviour must be assessed positively, unwanted pregnancies can still occur. The ninth survey of the Federal Centre for Health Education (Bundeszentrale für gesundheitliche Aufklärung, BZgA) on sexuality in the age group 14 to 25 years also provides information on which sources of information adolescents and young adolescents use.

The fact sheet by Prütz et al. shows, on the base of the data of the Federal Statistical Office, that although abortions continue to decline, the structure of care, which varies from region to region, is suboptimal. For example, the proportion of medical abortions is comparatively low, and the number of facilities performing abortions is still declining, so that women sometimes have to travel long distances – this is also an issue that requires attention from a public health perspective.

The studies of the RKI and the BZgA provide important indications for target group-oriented prevention and health communication. Even though this individual-based approach to increasing knowledge may be important, it is surprising from a public health perspective that structural conditions are largely ignored in the discussion about promoting sexual health and sexual well-being. Vulnerable groups in adolescence and young adulthood are the focus of an important model project of the Interdisciplinary Centre for Sexual Health and Medicine “WIR” (Walk in Ruhr), which is supported by the German Association of Private Health Insurers. The project aims to promote the sexual health of young people in challenging life situations – such as adolescents and young adults without a home or with addiction problems, imprisonment or pay-sex experiences. The aim of the programme “Young Worlds of Life (Junge Welten Leben, JuWeL)” is to establish a positive health-promoting sexual culture in the living environments of the target groups, for example in open prisons, in residential groups or counselling centres. In the field of sexual health, too, behavioural and institutional approaches need to go hand in hand – this is an exciting field of learning that we are facing.

The main topic in this issue of the Journal of Health Monitoring is also used to propose a way of assessing sex/gender in standardised surveys. Even before the amendment of the Civil Status Act in 2018, which now also allows an entry beyond the binary assignment into male/female, the recording of sex/gender was a challenge. The theoretical work of women’s (health) research on the differentiation between biological sex and social gender has demonstrated since the 1980s that the two categories do not have to coincide in the individual attribution. However, the question of how social gender can be recorded has remained unanswered until now, especially when only a few questions can be asked in representative studies with standardised survey instruments. The team of the RKI now presents a pragmatic proposal, which has already been tested in the GEDA 2019/2020-EHIS study and builds on international experiences: The sex/gender is asked in a two-step procedure. The question about which sex is registered on the birth certificate (for those currently included in the surveys in a binary format) is followed by a question about which gender the respondents feel they belong to (male/female/another, and that is). This offers the possibility to capture persons beyond the biological binary categorisation. At the same time, continuity with previous surveys is maintained because a binary evaluation option remains. The experiences of the interviewers reported in the article show that the respondents are irritated in some cases, but that this two-stage questioning basically works. Although this form of questionnaire may be too crude for well-founded analyses that take gender diversity into account, it is certainly a first step in the right direction when it is used for representative surveys.
